# Penicillin plus Ceftriaxone versus Ampicillin plus Ceftriaxone Synergistic Potential against Clinical Enterococcus faecalis Blood Isolates

**DOI:** 10.1128/spectrum.00621-22

**Published:** 2022-06-15

**Authors:** Jaclyn A. Cusumano, Ruhma Khan, Zeel Shah, Cassie Philogene, Amrita Harrichand, Vanthida Huang

**Affiliations:** a Department of Pharmacy Practice, Arnold & Marie Schwartz College of Pharmacy and Health Sciences, Brooklyn, New York, USA; b Department of Pharmaceutical Sciences, Arnold & Marie Schwartz College of Pharmacy and Health Sciences, Brooklyn, New York, USA; c Department of Pharmacy Practice, Midwestern University College of Pharmacy-Glendale Campus, Glendale, Arizona, USA; Hartford Hospital

**Keywords:** *Enterococcus faecalis*, ampicillin, ceftriaxone, penicillin, synergy

## Abstract

Penicillin plus ceftriaxone is a promising alternative to ampicillin plus ceftriaxone for the treatment of Enterococcus faecalis infective endocarditis. Limited data is available supporting the utilization of penicillin plus ceftriaxone. A total of 20 E. faecalis isolates; one wild-type strain (JH2-2) and 19 clinical blood strains were assessed for penicillin plus ceftriaxone and ampicillin plus ceftriaxone synergy using a 24-h time-kill experiment. Susceptibility was determined by broth microdilution. Differences in bactericidal, bacteriostatic, or inactivity, as well as synergy between treatments were assessed by chi-square or Fisher exact test. All E. faecalis isolates were considered susceptible to ampicillin and penicillin. Ampicillin plus ceftriaxone versus penicillin plus ceftriaxone similarly demonstrated synergy. Bactericidal activity was more commonly observed for ampicillin plus ceftriaxone versus penicillin plus ceftriaxone. Among isolates with a penicillin MIC of 4 μg/mL (*n* = 7), synergistic activity for both combinations was less common compared to isolates with a penicillin MIC ≤ 2 μg/mL (*n* = 13). Ampicillin plus ceftriaxone and penicillin plus ceftriaxone demonstrate similar synergistic potential against E. faecalis clinical blood isolates, but strains with higher penicillin and ceftriaxone MICs less frequently demonstrated synergy. Further research is warranted to determine the role of the penicillin plus ceftriaxone therapy and the penicillin MIC in clinical practice.

**IMPORTANCE** Penicillin plus ceftriaxone demonstrates similar synergistic activity against *Enterococcus faecalis* to ampicillin plus ceftriaxone. Isolates with a penicillin MIC of 4 mg/L and a ceftriaxone MIC of 512 or higher, lack penicillin plus ceftriaxone synergy despite the penicillin susceptibility MIC breakpoint of 8 mg/L.

## INTRODUCTION

Enterococcus faecalis is a leading cause of infective endocarditis (IE), with a greater than 30% mortality rate (1–4). Limited treatment options are available due to the intrinsic resistance of E. faecalis to the majority of available antibiotics (5). Penicillins (e.g., penicillin, ampicillin) are one of the few classes of antibiotics active against E. faecalis. In severe infections such as IE, a penicillin alone demonstrates only bacteriostatic activity and leads to an increased rate of treatment failure (6, 7). Therefore, combination therapy is essential to achieve synergistic, bactericidal activity against E. faecalis IE (6). A penicillin plus an aminoglycoside was the first combination utilized and significantly improved mortality (6). Due to rising aminoglycoside resistance and associated nephrotoxicity, ampicillin plus ceftriaxone (ampicillin+ceftriaxone) is emerging as a preferred alternative therapy (1, 6).

Despite E. faecalis intrinsic resistance to all cephalosporins including ceftriaxone, a synergistic relationship is observed with ampicillin and ceftriaxone via total saturation of penicillin-binding proteins (PBP) (7). Treatment of E. faecalis IE with ampicillin+ceftriaxone requires a 6-week course (6), which presents a challenge when coordinating outpatient parenteral antimicrobial therapy (OPAT) due to ampicillin stability. Historically, ampicillin was known to be stable (after reconstitution) for approximately 8 h at room temperature, which led to clinicians prescribing penicillin plus ceftriaxone due to improved penicillin stability (24 h at room temperature), despite limited evidence of primary support (8–11). Recent data demonstrates prolonged stability of ampicillin up to 30 h at room temperature (12, 13); however, ampicillin still lacks stability in an elastomeric pump, which is an advantageous outpatient intravenous medication delivery system that allows patients the freedom to travel with their medication in their pocket or a pouch without having to have the medication hung superiorly to the infusion pump (9). Penicillin maintains stability in an elastomeric pump (9), thus penicillin+ceftriaxone is a promising alternative to ampicillin+ceftriaxone.

Penicillin+ceftriaxone requires further evaluation as penicillin is known to have higher MICs compared to ampicillin against E. faecalis (14). There are also increasing reports of E. faecalis isolates with alterations in essential *pbp4* that demonstrate penicillin resistance but are ampicillin susceptible (3, 15). Clinical data is limited in supporting the utilization of penicillin+ceftriaxone (10, 11, 16, 17). A comparison of penicillin+ceftriaxone *in vitro* synergy to ampicillin+ceftriaxone has only been assessed in checkboard assays, which are limited due to the wide variability in result interpretation (17–19). Synergy assessment via *in vitro* time-kill experiments have not yet been reported. We hypothesized that penicillin+ceftriaxone will have equivalent *in vitro* synergy to ampicillin+ceftriaxone against E. faecalis blood isolates.

## RESULTS

### Susceptibility testing.

All isolates were considered susceptible to ampicillin and penicillin (Table 1). Wild-type isolate, JH2-2, had an ampicillin MIC of 0.5 μg/mL, which was lower than previously published MIC of 2 μg/mL (20). To confirm findings, we repeated the assay twice and obtained the same value. The penicillin MIC for JH2-2 was similar to previously published findings (15).

**TABLE 1:** Enterococcus faecalis isolates MICs (μg/mL)

	Ampicillin		Penicillin		Ceftriaxone
Isolates	Broth microdilution	Clinical microbiology lab	Broth microdilution	Clinical microbiology lab	Broth microdilution
JH2-2	0.5		2		256–512
e2003	1	<2	2	1	128–256
e2006	0.5	<2	2	2	16
e2011	1	<2	2	2	512
e2012	0.5	<2	2	2	256
e2014	1	<2	2	4	512
e2015	1	<2	2	1	512–1,024
e2017	1	<2	2	2	512–1,024
e2020	1	<2	2	2	128
e2025	1	<2	2	8	128
e2029	0.5	<2	2	4	128
e2031	0.5	<2	1	2	128
e2032	0.5	<2	2	2	256–512
e2008	1	<2	4	8	2,048
e2009	1	<2	4	8	2,048
e2010	2	<2	4	8	512
e2018	1	<2	4	8	>2048
e2024	1	<2	4	4	256
e2027	1	<2	4	16	>2,048
e2028	1	<2	4	16	>2,048

All ampicillin MICs were concordant with the clinical microbiology laboratory results. Penicillin MICs were within one to two, 2-fold dilutions of the reported MIC from the clinical microbiology laboratory. A total of 13 isolates had a penicillin MIC ≤ 2 μg/mL and seven isolates had a penicillin MIC of 4 μg/mL. All ceftriaxone MICs were elevated as expected due to intrinsic resistance, except one isolate that had an MIC of 16 μg/mL (e2006). Most isolates with a penicillin MIC of 4 μg/mL had a ceftriaxone MIC ≥ 2048 μg/mL (*n* = 5/7, 71%) compared to most isolates with a penicillin MIC of 2 μg/mL had a ceftriaxone MIC ≤ 512 μg/mL (*n* = 11/13, 85%) (Table 1).

### Time-kill assays.

All isolates against ampicillin or penicillin monotherapy at 0.25xMIC demonstrated inactivity (Table 2). Ceftriaxone monotherapy also demonstrated inactivity against all isolates, except one isolate which was bacteriostatic (e2006; −0.44 ± 0.54 log_10_ CFU/mL). Ampicillin versus penicillin monotherapy at 0.5xMIC less commonly demonstrated inactivity (*n* = 13, 65% versus *n* = 19, 95%; *P* = 0.04). Ampicillin versus penicillin at 1xMIC similarly did not demonstrate inactivity (*n* = 4, 20% versus *n* = 2, 10%, *P* = 0.66), with majority of isolates demonstrating bactericidal activity against ampicillin (*n* = 11, 55%) and majority of isolates demonstrating bacteriostatic activity against penicillin (*n* = 11, 55%).

**TABLE 2:** Beta-lactam monotherapies against E. faecalis: Time-kill assay change in log_10_ CFU/mL from the initial inoculum at 24 h

	Ceftriaxone alone	Ampicillin alone			Penicillin alone		
Isolates	17.2 mcg/mL	0.25 × MIC	0.5 × MIC	1 × MIC	0.25 × MIC	0.5 × MIC	1 × MIC
Isolates with penicillin MIC ≤ 2 mcg/mL							
JH2-2	+1.94 ± 0.08	+2.05 ± 0.26	+1.81 ± 0.18	−0.08 ± 0.18^*b*^	+2.09 ± 0.06	+1.84 ± 0.22	−1.26 ± 0.43^*b*^
e2003	+1.62 ± 0.13	+2.06 ± 0.16	+0.83 ± 0.37	−4.05 ± 0.00^*a*^	+2.02 ± 0.08	+0.50 ± 0.10	−3.84 ± 0.30^*a*^
e2006	−0.44 ± 0.54^*b*^	+1.97 ± 0.03	+1.81 ± 0.37	−3.31 ± 0.08^*a*^	+2.04 ± 0.09	+1.98 ± 0.06	−3.91 ± 0.11^*a*^
e2011	+2.52 ± 0.45	+2.21 ± 0.07	+1.28 ± 0.04	−4.14 ± 0.00^*a*^	+2.23 ± 0.21	+2.08 ± 0.21	+0.23 ± 0.22
e2012	+1.08 ± 0.12	+1.95 ± 0.06	+1.58 ± 0.04	+0.16 ± 0.06	+1.93 ± 0.06	+1.32 ± 0.21	−3.23 ± 0.31^*a*^
e2014	+1.63 ± 0.11	+1.75 ± 0.03	+0.1 ± 0.18	−2.02 ± 0.05^*b*^	+2.00 ± 0.10	+1.43 ± 0.01	−1.90 ± 0.99^*b*^
e2015	+1.67 ± 0.06	+1.57 ± 0.07	−0.58 ± 0.51^*b*^	−4.12 ± 0.00^*a*^	+1.99 ± 0.16	+1.48 ± 0.08	−2.73 ± 0.12^*b*^
e2017	+1.72 ± 0.06	+1.88 ± 0.08	−0.05 ± 0.39^*b*^	−2.62 ± 0.54^*b*^	+1.20 ± 0.10	+1.38 ± 0.15	−2.58 ± 0.02^*b*^
e2020	+1.37 ± 0.12	+1.81 ± 0.12	+1.23 ± 0.28	−4.09 ± 0.00^*a*^	+2.05 ± 0.06	+1.61 ± 0.02	−3.04 ± 1.07^*a*^
e2025	+1.27 ± 0.07	+1.81 ± 0.12	+0.90 ± 0.01	−4.25 ± 0.05^*a*^	+1.65 ± 0.14	+1.21 ± 0.02	−0.60 ± 0.20^*b*^
e2029	+1.59 ± 0.01	+2.26 ± 0.21	+1.77 ± 0.01	+0.54 ± 0.04	+1.98 ± 0.03	+0.60 ± 0.00	−3.36 ± 0.76^*a*^
e2031	+1.98 ± 0.12	+1.81 ± 0.11	−2.86 ± 0.45^*b*^	−4.11 ± 0.00^*a*^	+2.24 ± 0.07	+0.95 ± 0.34	−3.23 ± 0.05^*a*^
e2032	+1.27 ± 0.07	+1.82 ± 0.06	+1.50 ± 0.00	+0.96 ± 0.01	+1.62 ± 0.17	+0.44 ± 0.15	−2.04 ± 1.07^*b*^
Isolates with penicillin MIC of 4 mcg/mL							
e2008	+2.11 ± 0.03	+0.34 ± 0.05	−3.00 ± 0.14^*a*^	−3.32 ± 0.05^*a*^	+1.03 ± 0.03	+0.68 ± 0.13	−2.69 ± 0.03^*b*^
ve2009	+2.21 ± 0.13	+1.06 ± 0.16	−2.02 ± 0.09^*b*^	−4.08 ± 0.00^*a*^	+1.96 ± 0.12	+1.35 ± 0.04	+0.09 ± 0.14
e2010	+2.05 ± 0.07	+1.46 ± 0.05	−2.64 ± 0.25^*b*^	−2.73 ± 0.08^*b*^	+1.72 ± 0.04	+1.00 ± 0.10	−0.28 ± 0.04^*b*^
e2018	+2.42 ± 0.02	+1.87 ± 0.01	+1.46 ± 0.04	−1.98 ± 0.09^*b*^	+2.22 ± 0.22	+1.75 ± 0.06	−1.24 ± 0.15^*b*^
e2024	+1.61 ± 0.04	+2.05 ± 0.04	+1.23 ± 0.10	−3.34 ± 0.02^*a*^	+1.78 ± 0.00	−0.67 ± 0.81^*b*^	−3.83 ± 0.12^*a*^
e2027	+2.08 ± 0.04	+1.58 ± 0.11	+1.82 ± 0.01	+0.29 ± 0.28	+1.81 ± 0.23	+1.31 ± 0.03	−1.80 ± 0.00^*b*^
e2028	+2.11 ± 0.12	+1.34 ± 0.06	−0.20 ± 0.16^*b*^	−3.51 ± 0.58^*a*^	+1.97 ± 0.00	+1.63 ± 0.00	−1.27 ± 0.23^*b*^

a Bactericidal activity was observed, defined as ≥3-log_10_ decrease in CFU/mL from initial inoculum.

b Bacteriostatic activity was observed, defined as <3-log_10_ decrease in CFU/mL from initial inoculum.

Combination ampicillin+ceftriaxone versus penicillin+ceftriaxone similarly demonstrated synergy at 0.25×, 0.5×, and 1 × MIC (*n* = 9, 45% versus *n* = 7, 35%, *P* = 0.52; *n* = 11, 55% versus *n* = 12, 60%, *P* = 0.75; and *n* = 5, 25% versus *n* = 5, 25%, *P* = 1.00; respectively) (Table 3). Bactericidal activity was more commonly observed for ampicillin+ceftriaxone versus penicillin+ceftriaxone at 0.25 × MIC (*n* = 9, 45% versus *n* = 2, 10%, *P* = 0.01). Inactivity was less commonly observed for ampicillin+ceftriaxone versus penicillin+ceftriaxone at 0.5 × MIC (*n* = 1, 5% versus *n* = 7, 35%, *P* = 0.04). All other antibacterial activity was similar between the two combinations.

**TABLE 3:** Beta-lactam combination therapies against E. faecalis: Time-kill assay change in log_10_ CFU/mL from the initial inoculum at 24 h

	Ampicillin + ceftriaxone			Penicillin + ceftriaxone		
Isolate	0.25 × MIC	0.5 × MIC	1 × MIC	0.25 × MIC	0.5 × MIC	1 × MIC
Isolates with penicillin MIC ≤ 2 mcg/mL						
JH2-2	−2.04 ± 0.12^*b*^^,^^*c*^	−4.13 ± 0.00^*a*^^,^^*c*^	−4.13 ± 0.00^*a*^^,^^*c*^	+1.12 ± 0.08	+0.45 ± 0.82	−3.01 ± 0.08*^a^*
e2003	−4.05 ± 0.00*^a^*	−4.05 ± 0.00*^a^*	−4.05 ± 0.00*^a^*	+1.90 ± 0.17	−3.7 ± 0.25^*a*^^,^^*c*^	−4.05 ± 0.00*^a^*
e2006	−4.05 ± 0.00*^a^*	−4.05 ± 0.00*^a^*	−4.05 ± 0.00*^a^*	−2.96 ± 0.12^*b*^^,^^*c*^	−4.05 ± 0.00^*a*^^,^^*c*^	−4.05 ± 0.00*^a^*
e2011	−3.68 ± 0.09^*a*^^,^^*c*^	−4.14 ± 0.00^*a*^^,^^*c*^	−4.14 ± 0.00*^a^*	+1.60 ± 0.23	−4.11 ± 0.05^*a*^^,^^*c*^	−3.95 ± 0.10^*a*^^,^^*c*^
e2012	+1.20 ± 0.32	−4.01 ± 0.00^*a*^^,^^*c*^	−4.01 ± 0.00^*a*^^,^^*c*^	+1.02 ± 0.25	−3.94 ± 0.00^*a*^^,^^*c*^	−3.92 ± 0.12*^a^*
e2014	−4.05 ± 0.00^*a*^^,^^*c*^	−4.05 ± 0.00^*a*^^,^^*c*^	−4.05 ± 0.00^*a*^^,^^*c*^	−0.38 ± 0.24^*b*^^,^^*c*^	−3.98 ± 0.00^*a*^^,^^*c*^	−4.05 ± 0.00^*a*^^,^^*c*^
e2015	−4.12 ± 0.00^*a*^^,^^*c*^	−4.12 ± 0.00^*a*^^,^^*c*^	−4.12 ± 0.00*^a^*	−1.2 ± 0.12^*b*^^,^^*c*^	−2.2 ± 0.29^*b*^^,^^*c*^	−4.12 ± 0.00*^a^*
e2017	−2.31 ± 0.16^*b*^^,^^*c*^	−3.99 ± 0.00^*a*^^,^^*c*^	−3.99 ± 0.00*^a^*	−2.84 ± 0.04^*b*^^,^^*c*^	−2.61 ± 0.11^*b*^^,^^*c*^	−3.99 ± 0.00*^a^*
e2020	−4.09 ± 0.00^*a*^^,^^*c*^	−4.09 ± 0.00^*a*^^,^^*c*^	−4.09 ± 0.00*^a^*	−0.16 ± 0.55^*b*^	−4.06 ± 0.05^*a*^^,^^*c*^	−4.09 ± 0.00*^a^*
e2025	−3.89 ± 0.00^*a*^^,^^*c*^	−4.28 ± 0.00^*a*^^,^^*c*^	−4.28 ± 0.00*^a^*	−2.01 ± 0.14^*b*^^,^^*c*^	−4.06 ± 0.00^*a*^^,^^*c*^	−4.28 ± 0.00^*a*^^,^^*c*^
e2029	+1.73 ± 0.01	−3.75 ± 0.45^*a*^^,^^*c*^	−3.44 ± 0.89^*a*^^,^^*c*^	+0.97 ± 0.68	−1.57 ± 0.71^*b*^^,^^*c*^	−4.07 ± 0.00*^a^*
e2031	−3.16 ± 0.44^*a*^^,^^*c*^	−4.11 ± 0.00*^a^*	−4.11 ± 0.00*^a^*	−4.05 ± 0.09^*a*^^,^^*c*^	−3.76 ± 0.25^*a*^^,^^*c*^	−4.11 ± 0.00*^a^*
e2032	+1.63 ± 0.12	−2.49 ± 0.09^*b*^	−4.14 ± 0.00*^a^*	+1.82 ± 0.24	−0.05 ± 0.19^*b*^	−4.14 ± 0.00^*a*^^,^^*c*^
Isolates with penicillin MIC of 4 mcg/mL						
e2008	−0.40 ± 0.03^*b*^	−2.56 ± 0.12^*b*^	−3.44 ± 0.04*^a^*	+1.00 ± 0.07	+0.46 ± 0.10	−2.5 ± 0.04^*b*^
e2009	−0.57 ± 0.25^*b*^	−2.61 ± 0.07^*b*^	−4.08 ± 0.00*^a^*	+1.73 ± 0.09	+1.12 ± 0.19	−2.15 ± 0.20^*b*^^,^^*c*^
e2010	+0.19 ± 0.06	−1.51 ± 0.06^*b*^	−3.35 ± 0.10*^a^*	+1.00 ± 0.08	+0.70 ± 0.03	−0.82 ± 0.00^*b*^
e2018	+1.46 ± 0.00	+0.4 ± 0.02	−3.37 ± 0.07*^a^*	+1.97 ± 0.23	+1.66 ± 0.05	−1.49 ± 0.06^*b*^
e2024	−3.42 ± 0.71^*a*^^,^^*c*^	−3.92 ± 0.00^*a*^^,^^*c*^	−3.92 ± 0.00*^a^*	−3.51 ± 0.02^*a*^^,^^*c*^	−3.92 ± 0.00^*a*^^,^^*c*^	−3.92 ± 0.00*^a^*
e2027	+1.18 ± 0.19	−0.31 ± 0.13^*b*^^,^^*c*^	−2.75 ± 0.44^*b*^^,^^*c*^	+1.66 ± 0.02	+0.95 ± 0.24	−2.12 ± 0.11^*b*^
e2028	+1.26 ± 0.20	−1.21 ± 0.33^*b*^	−3.64 ± 0.18*^a^*	+1.99 ± 0.03	+1.56 ± 0.08	−1.27 ± 0.25^*b*^

a Bactericidal activity was observed, defined as ≥3-log_10_ decrease in CFU/mL from initial inoculum.

b Bacteriostatic activity was observed, defined as <3-log_10_ decrease in CFU/mL from initial inoculum.

c Synergy was detected as indicated by gray shading, defined as ≥2-log_10_ decrease in CFU/mL at 24 h from the most active single agent was observed.

Among isolates with a penicillin MIC of 4 μg/mL (*n* = 7), synergistic activity for ampicillin+ceftriaxone and penicillin+ceftriaxone was less common compared to isolates with a penicillin MIC ≤ 2 μg/mL (*n* = 13) (Table 3). Bactericidal activity was also more common among isolates with a penicillin MIC ≤ 2 versus a penicillin MIC of 4 μg/mL. Only one isolate (e2024) demonstrated bactericidal activity and synergy at 0.25× and 0.5 × MIC for both combinations and was the only penicillin MIC of 4 μg/mL isolate with a lower ceftriaxone MIC of 256 μg/mL. All other isolates with a penicillin MIC of 4 μg/mL exposed to penicillin+ceftriaxone at 0.25× and 0.5 × MIC were inactive and had a higher ceftriaxone MIC (range 512 to >2,048 μg/mL).

## DISCUSSION

Among 20 clinical Enterococcus faecalis blood isolates, similar synergistic activity was observed for ampicillin+ceftriaxone and penicillin+ceftriaxone combinations. More frequent bactericidal activity was demonstrated among isolates treated with ampicillin+ceftriaxone versus penicillin+ceftriaxone. Penicillin+ceftriaxone also demonstrated more bactericidal at higher penicillin concentrations. Ampicillin+ceftriaxone and penicillin+ceftriaxone synergy was less frequently observed among isolates with a higher penicillin MIC of 4 μg/mL as well as higher ceftriaxone MICs. The observed differences in activity may impact clinical outcomes in patients with E. faecalis IE, especially since β-lactam-based treatment failure has been shown to occur more frequently in patients infected with a penicillin-resistant ampicillin-susceptible E. faecalis (3). According to CLSI, the clinical MIC breakpoint for penicillin and E. faecalis is ≤ 8 μg/mL, indicating our isolates were still susceptible (21). Interestingly, majority of our isolates with a penicillin MIC of 4 μg/mL had a higher reported MIC by Vitek 2 (Table 1), with two isolates having an MIC above the breakpoint (i.e., 16 μg/mL). Our findings of lower MICs by broth microdilution compared to Vitek 2 are similar to a previous report of 49 penicillin-resistant ampicillin-susceptible E. faecalis isolates, which found that 93.9% of isolate MICs by Vitek 2 were two, 2-fold dilutions higher than broth microdilution (22).

While the prevalence of penicillin-resistant ampicillin-susceptible E. faecalis remains unknown in the Unites States (US), β-lactam-based treatments remain the standard of care. Ampicillin+ceftriaxone is the most commonly used first-line combination to treat E. faecalis IE due to the improved safety profile compared to aminoglycoside-based treatments as well as rising aminoglycoside resistance up to 60% (1, 6). However, due to the challenges of coordinating ampicillin therapy in the outpatient setting patients often receive penicillin+ceftriaxone (8, 9). Limited clinical data is available supporting the use of penicillin+ceftriaxone in clinical practice for E. faecalis IE and has only been assessed in patients who have already received standard of care treatment. The first discussion was a retrospective review in the US that identified five patients who were discharged on penicillin+ceftriaxone after receiving 3 to 8 days of ampicillin+ceftriaxone inpatient (11). Two of the five patients were lost to follow-up and the other three achieved clinical cure with no relapse at 90 days (11). The penicillin MICs were not reported in this study (11). Another case series of four patients in the US who received penicillin+ceftriaxone, after already receiving standard of care [i.e., ampicillin+ceftriaxone (*n* = 3) and penicillin plus gentamicin (*n* = 1)] treatment for a range of 3 to 32 days, reported no recurrence in infection at 6 months (10). The authors reported penicillin MICs for three of the four patients by Etest, where two patients had an MIC of 4 μg/mL, and one had an MIC of 2 μg/mL (10). Etest methodology is found to correlate well to broth microdilution methodology, which perhaps suggests that a penicillin MIC of 4 μg/mL does not always indicate treatment failure as we observed with our isolates (22). However, one of the patients received chronic amoxicillin oral suppression and the other patient received penicillin+gentamicin for 32 days prior to transitioning to penicillin+ceftriaxone, which may falsely make penicillin+ceftriaxone appear efficacious.

A larger multicenter case series in New Zealand of 41 patients with enterococcal endocarditis (E. faecalis, *n* = 40 and Enterococcus faecium, *n* = 1) compared outpatient treatment with penicillin plus gentamicin (*n* = 20) versus penicillin+ceftriaxone (*n* = 23), found no difference in recurrence (11% versus 5%, *P* = 0.59) but a greater incidence of side effects in patients receiving gentamicin therapy (35% versus 0%, *P* < 0.01) (16). Patients received a median of 15 days (interquartile range [IQR] 9–18.5 days) of inpatient penicillin, amoxicillin, amoxicillin-clavulanic acid, piperacillin, or piperacillin-tazobactam plus a synergy antibiotic (i.e., ceftriaxone or gentamicin) prior to discharge (16). The predominant synergy antibiotic received during the first 14 days of treatment was then selected for the patient on discharge (16). Penicillin susceptibility was available for 32 of the isolates, with a median MIC of 3 (IQR 2–4), but methodology was not reported (16). Similar efficacy between the two combinations may be due to more patients in the penicillin+ceftriaxone group receiving chronic amoxicillin oral suppression (5% versus 35%, *P* = 0.02) (16).

Most recently, a single-center retrospective cohort study in Australia identified 20 patients with an E. faecalis endovascular infection who received penicillin+ceftriaxone via OPAT from their existing OPAT database (17). Six patients (30%) experienced an unplanned readmission, one patient (5%) had a relapse in bacteremia within 6 months, and 1-year mortality was 15% (17). All isolates were considered susceptible to penicillin by Vitek 2, but MICs were not reported (17). A random six isolates were selected for testing by broth microdilution and synergy assessment by checkerboard, which revealed a median penicillin MIC of 1 μg/mL (IQR 0.5–1 μg/mL) and synergy for four isolates against ampicillin+ceftriaxone and three isolates against penicillin+ceftriaxone (17). It is important to note, however, that the interpretation of the fractional inhibitory concentration index (FICI) was not indicated (17). Overall, the clinical data is limited, and randomized controlled trials are needed to determine the equivalence of penicillin+ceftriaxone to ampicillin+ceftriaxone and the role of the E. faecalis penicillin MIC in predicting treatment success.

Only one other *in vitro* study has been published to date, which also compared checkboard synergy of ampicillin+ceftriaxone versus penicillin+ceftriaxone among 28 clinical E. faecalis blood isolates from Germany and one wild-type isolate (ATCC 29212) (19). The ceftriaxone concentrations utilized included the free plasma trough concentrations for a 2 g IV q12h, 4 g IV q24h, and 2 g IV q24h regimens, which were 4, 1.5, and 1 μg/L, respectively (19). Conversely, we utilized a free steady-state plasma concentration of 17.2 μg/mL based on clinical pharmacokinetic data for a 2 g IV q12h regimen, where the extrapolated trough would be 9.13 μg/mL versus the 4 μg/mL utilized by Thieme et al. (19, 23). The difference in concentration is likely due to the variability in patient pharmacokinetics but should be considered when interpreting the *in vitro* synergy results (23–25). Additionally, the authors utilized three different definitions for the FICI, which leads to wide variability in result interpretation (18, 19). When utilizing an FICI ≤ 0.5 to indicate synergy a total of 22 (75.9%) and 16 (55.2%) isolates were synergistic against ampicillin+ceftriaxone and penicillin+ceftriaxone, respectively (19). When utilizing the median FICI of 0.8 as the synergy threshold, an additional five isolates (*n* = 21, 72.4%) demonstrated penicillin+ceftriaxone synergy and no additional isolates demonstrated ampicillin+ceftriaxone synergy (19). We observed comparable rates of synergy between the two combinations. We also observed minimal synergy for both combinations in isolates with a penicillin MIC of 4 μg/mL, whereas Thieme et al. found a strong inverse correlation indicating that the higher the penicillin MIC the lower the FICI (r_s_ = -0.61, *P* = 0.001) (19). However, these results are difficult to interpret as the lower the FICI does not necessarily mean more synergy.

In addition, among our isolates with a penicillin MIC of 4 μg/mL, the ceftriaxone MICs were higher compared to isolates with a penicillin MIC ≤ 2 μg/mL. One isolate with a penicillin MIC of 4 μg/mL (e2024) had a ceftriaxone MIC of 256 μg/mL and was also the only isolate with a penicillin MIC of 4 μg/mL that demonstrated synergy against both ampicillin+ceftriaxone and penicillin+ceftriaxone. Similarly, Thieme L et al.’s *in vitro* checkboard study found that isolates with a ceftriaxone MIC > 1024 μg/mL (*n* = 4) did not demonstrate synergy for either combination, and ceftriaxone concentrations required to reduce the ampicillin or penicillin were frequently unachievable or higher than physiologically achievable ceftriaxone concentrations (19). Therefore, we chose to utilize the free plasma steady-state concentrations (*fCpss*) of ceftriaxone to improve the clinical applicability of our results similar to previous work (26). Utilization of ceftriaxone at 0.12×, 0.25×, and 0.5 × MIC in a time-kill assay in previous work with isolate JH2-2 (ceftriaxone MIC 512 μg/mL) also yielded similar synergistic results (27). The relationship of the ceftriaxone MIC along with the penicillin MIC to β-lactam synergistic potential may be related to changes in essential penicillin-binding protein-4 (PBP4). Although alterations in *pbp4* have only been reported in penicillin-resistant ampicillin-susceptible isolates (15), further investigation is warranted to determine if *pbp4* mutations are present in isolates with higher penicillin MICs near the breakpoint (i.e., 4 and 8 μg/mL).

The strength of our study was the inclusion of clinical blood E. faecalis strains and utilization of time-kill assays which have a clear synergy definition compared to checkboard methodology. While time-kill assays are superior to checkboard methodology, our results are limited by the static nature of these assays, which limits the applicability to determine appropriate dosing that maximizes bactericidal activity. To improve applicability to patient care we utilized physiologic concentrations of ceftriaxone, but unfortunately the physiologic steady-state plasma concentrations for ampicillin and penicillin were above the MIC and would eradicate the organism without ceftriaxone in combination. As a result, we utilized subinhibitory concentrations at 0.25×, 0.5×, and 1 × MIC, which led to variability in the concentrations utilized across the isolates. Further research is warranted to determine optimal penicillin+ceftriaxone dosing and the role of the E. faecalis penicillin MIC in predicting treatment success.

### Conclusion.

Overall, ampicillin+ceftriaxone and penicillin+ceftriaxone demonstrates similar synergistic potential but ampicillin+ceftriaxone is more bactericidal. Strains with higher penicillin and ceftriaxone MICs less frequently demonstrated synergy with both ampicillin+ceftriaxone and penicillin+ceftriaxone. Higher penicillin concentrations were warranted to achieve bactericidal activity, but further research is warranted to determine the appropriate dose to optimize penicillin exposure.

## MATERIALS AND METHODS

### Bacterial isolates.

A total of 20 E. faecalis isolates were included; one wild-type strain (JH2-2) and 19 clinical strains from blood. Most clinical isolates obtained were ampicillin and penicillin susceptible by the clinical microbiology laboratory (Vitek 2, bioMérieux, Inc., Durham, NC). There were two isolates that had a penicillin MIC of 16 μg/mL, which is one 2-fold dilution above the clinical breakpoint for penicillin is ≤ 8 μg/mL (21). All isolates were stored in (CryoCare, Stamford, TX; tryptic soy broth plus glycerol) at −80°C and were subcultured once on brain heart infusion agar for 18–24 h at 35°C prior to each experiment.

### Antimicrobials and media.

Antibiotic powders were purchased from Sigma-Aldrich, Inc. (St. Louis, MO): ampicillin sodium (product number: A0166), penicillin G potassium salt (product number: 46609), and ceftriaxone sodium (product number: PHR1382). Experiments were performed using cation adjusted (calcium, 25 μg/mL; magnesium, 12.5 μg/mL) Mueller-Hinton broth (MHB; BD Difco, Sparks, MD). All viable cell count samples and subcultures were plated on brain heart infusion agar (BHIA; BD Difco, Sparks, MD) (15, 28, 29).

### Susceptibility testing.

MICs were performed for ampicillin, penicillin, and ceftriaxone by broth microdilution to confirm clinical microbiology laboratory results according to CLSI (21). All MICs were performed in duplicate and were repeated to confirm any discordant MIC values between the clinical microbiology laboratory and our laboratory.

### Time-kill assays.

A 24-h time-kill experiment was utilized to detect synergy for ampicillin+ceftriaxone versus penicillin+ceftriaxone against all 20 isolates. Experiments were performed in duplicate in a 12-well plate with a final volume of 2 mL. Assays were repeated to confirm findings if a wide standard deviation in results was observed. The starting inoculum was 10^6^ CFU/mL and plates were placed in the incubator at 35°C at 50 rotations per minute (rpm). Subinhibitory concentrations (0.25× and 0.5 × MIC) of ampicillin and penicillin were utilized as previously described (28), and 1xMIC was also tested. Ceftriaxone was tested at the free plasma steady-state concentration (*fCpss *=* *17.2 μg/mL) based on population pharmacokinetic data for a 2 g IV q12h regimen (t_1/2_ = 7.2 h, *f*C_max_ = 28.9 μg/mL), as subinhibitory concentrations would not be physiologically achievable due to the intrinsic resistance of ceftriaxone to enterococcus (23, 30). Each drug was tested as monotherapy and both ampicillin and penicillin were combined with ceftriaxone. Samples were obtained at 0, 4, and 24 h and diluted 1:10 in normal saline to obtain viable cell counts. Three 20 mcL samples of each dilution were plated onto BHIA and incubated for 18–24 h at 35°C, where the average of the three samples was taken to obtain a log_10_ CFU/mL viable cell count. Samples were directly obtained from the 12-well plate if bacterial growth was not visible for a lower limit of detection of 2-log_10_ CFU/mL. Antimicrobial activity was defined as bacteriostatic or bactericidal, which were defined as < 3-log_10_ CFU/mL or ≥ 3-log_10_ CFU/mL decrease from initial inoculum at 24 h. Inactivity was defined as an increase in log_10_ CFU/mL from initial inoculum at 24 h. Combination therapy activity was determined to be synergistic if a ≥ 2-log_10_ decrease in CFU/mL at 24 h from the most active single agent was observed (18).

### Statistical analysis.

Differences in bactericidal, bacteriostatic, or inactivity between ampicillin and penicillin monotherapies, and ampicillin+ceftriaxone and penicillin+ceftriaxone combinations were assessed by chi-square or Fisher exact test. Differences in synergy between ampicillin+ceftriaxone and penicillin+ceftriaxone, as well as between isolates with a penicillin MIC ≤ 2 μg/mL and isolates with a penicillin MIC of 4 μg/mL for each combination were assessed by chi-square or Fisher exact test. All statistical analyses were performed using R (version 4.1.2).
